# Characteristics and properties of a polysaccharide isolated from *Wolfiporia cocos* as potential dietary supplement for IBS

**DOI:** 10.3389/fnut.2023.1119583

**Published:** 2023-03-27

**Authors:** Xuan Yang, Shun Lu, Yuhan Feng, Chongjiang Cao, Yanliang Zhang, Shujie Cheng

**Affiliations:** ^1^Department of Food Nutrition and Safety/National R&D Center for Chinese Herbal Medicine Processing Technology, School of Engineering, China Pharmaceutical University, Nanjing, China; ^2^Nanjing Hospital of Chinese Medicine Affiliated to Nanjing University of Chinese Medicine, Nanjing, China; ^3^Nanjing Research Center for Infectious Diseases of Integrated Traditional Chinese and Western Medicine, Nanjing, China

**Keywords:** *Wolfiporia cocos*, polysaccharide, *in vitro* digestion, *in vitro* fermentation, gut microbiota, irritable bowel syndrome, diet therapy

## Abstract

**Introduction:**

As low FODMAP (Fermentable oligosaccharides, disaccharides, monosaccharides and polyols) diet therapy is recommended for most of Irritable Bowel Syndrome (IBS) patients, the consequent insufficient of dietary fibers (DFs) intake exert an adverse impact on intestinal health. It is necessary to find suitable DFs for IBS patients.

**Methods:**

This study extracted a water-insoluble polysaccharide from *Wolfiporia cocos* (WIP) by alkali-extraction and acid-precipitation method. Its molecular weight was detected by high performance gel permeation chromatography (HPGPC) analysis. The structure of WIP was analyzed by Fourier transform infrared (FT-IR) spectrum, Nuclear Magnetic Resonance (NMR) spectra and X-ray diffraction (XRD). The properties related to stability, digestion, viscosity, osmotic activity, adsorption and fermentation were investigated, aimed to explore the feasibility of WIP as a new DF supplement for patients with IBS. In addition, 16S rRNA sequencing analysis was conducted to explore its effects on IBS-related gut microbiota.

**Results and Discussion:**

The results showed that WIP had a single homogeneous composition and the molecular weight was 8.1 × 10^3^ Da. WIP was indicated as a kind of pyranose form with β anomeric configuration and the main chain of WIP was 1,3-β-glucan with amorphous structure. In addition to good thermal stability, WIP also has low bioavailability and can reach the colon mostly without being digested. Moreover, the low viscosity and osmotic activity, the high water- swelling and water/oil-holding capacity, fructose adsorption capacity and poor fermentation performance of WIP demonstrated that it is suitable for IBS patients. It is worth noting that WIP regulates IBS associated gut microbiota effectively, such as the abundance of Lachnospiraceae and Prevotella. These findings provide a theoretical basis for the development of WIP as a dietary supplement for IBS patients with low FODMAP diet therapy.

**GRAPHICAL ABSTRACT:**
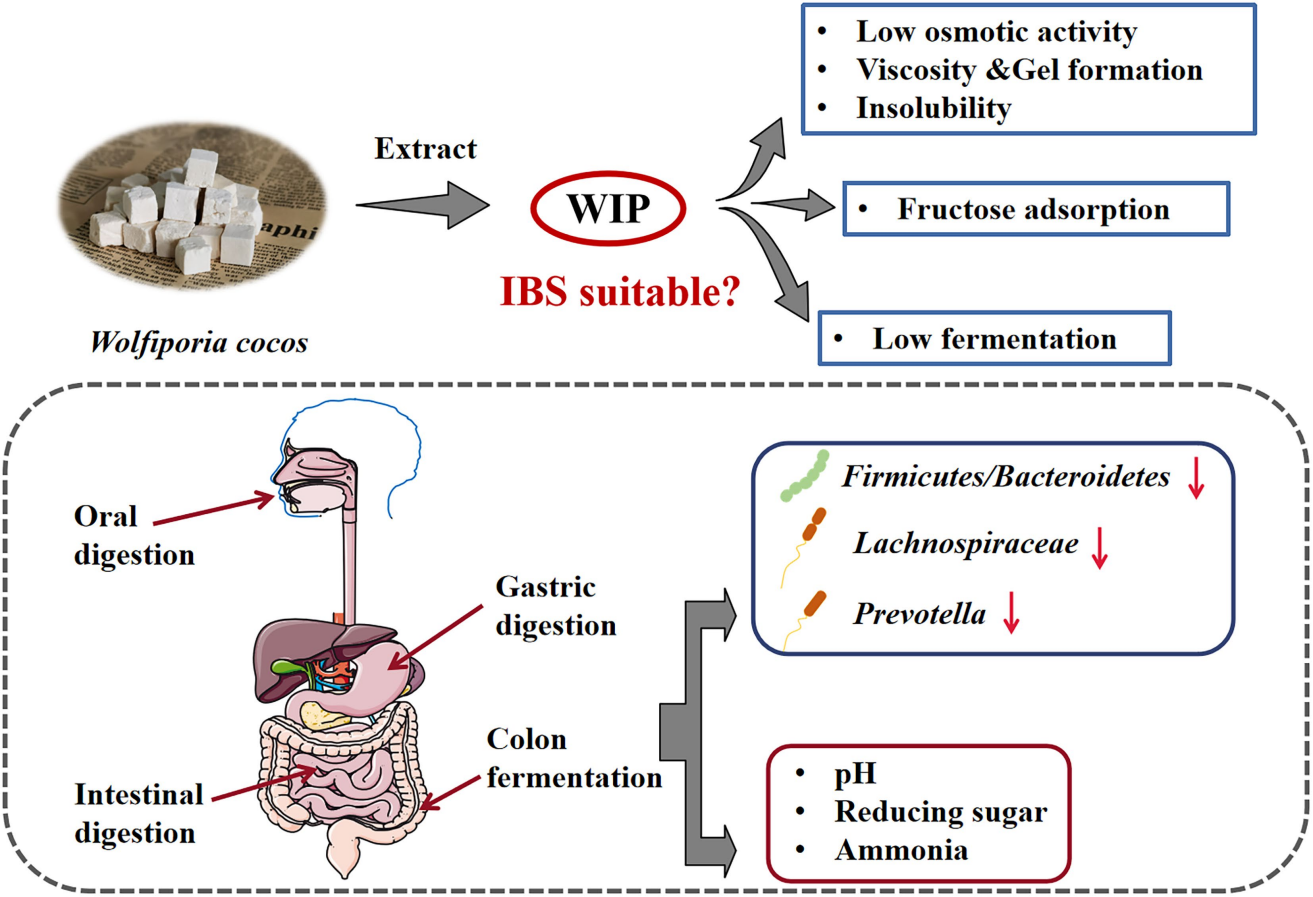

## 1. Introduction

*Wolfiporia cocos* (previously named as *Poria cocos*) has been used in Asia for more than 2000 years as a kind of edible-medicinal mushroom (1–3). It is reported that polysaccharide and triterpenoids are the main ingredients in the dried sclerotia of *W. cocos* (1, 4–6). Our previous study found that one of the triterpenoids from *W. cocos*, 16α-Hydroxytrametenolic Acid, enhances the function of intestinal barrier through glucocorticoid receptor-mediated PI3K/Akt/NF-κB signaling pathway (7). Polysaccharides from edible fungus have various biological activities, such as improving functional constipation and immune regulation (8). However, the direct utilization of natural polysaccharide in the *W. cocos*, which up to 80% in the dried sclerotia, is limited due to its water-insoluble property. Liu et al. demonstrated that oral administration of water-insoluble polysaccharide from *W. cocos* (WIP) can effectively improve glucose and lipid metabolism, reduce liver inflammation and steatosis in mice by regulating the gut microbiota (9, 10). Therefore, we speculate that WIP may be used as a functional dietary fiber (DF) to prevent intestinal disease and improve gut health.

Irritable bowel syndrome (IBS) is one of the most common functional gastrointestinal (GI) disorders, and its global prevalence is estimated to be 11.2% (11, 12). It is characterized by pain or abdominal discomfort accompanied with changes of bowel habits, which exerts significant impact on patients’ daily life (13, 14). Due to the accelerated pace of life and persistent mental stress, the number of IBS patients continues to increase in recent years. Treatment options for IBS include psychotherapy, medication, and dietary modification (15). However, a large number of psychological counseling will bring huge economic pressure to patients, and the effect of drug treatment is not obvious and there are many adverse reactions. In this case, diet therapy is becoming more and more popular. FODMAPs (fermentable oligosaccharides, disaccharides, monosaccharides, and polyols) describes carbohydrates that are neither digested nor absorbed in the human gut, including lactose, fructose in excess of glucose, sugar polyols (mannose and sorbitol), fructans and galactooligosaccharides (GOS, stachyose and raffinose) (16). Due to its high fermentable property and osmotic activity, FODMAPs can exacerbate symptoms of IBS patients (17). Therefore, low FODMAP diet is widely recommended by physicians as a first-line therapy for IBS patients (18). However, the concomitant problem with a low FODMAPs diet is a greatly reduction of DFs intake (19, 20), which aggravates intestinal problems such as constipation and gut microbiota disturbance, increases intestinal sensitivity, and is not conducive to the recovery of patients (21–24).

The application and therapeutic value of DFs depend on their functional properties. These properties determine that different DFs have specific effects on the gastrointestinal tract, including the formation of viscous gels, the expansion of fecal volume, and the impact on intestinal microbiota (25). In addition to the swelling property and water holding capacity, Atzler et al. pointed out that DF supplements suitable for IBS patients with low FODMAP diet therapy should meet four characteristics: insolubility, viscosity and gel formation, low osmotic activity and low fermentation (19). However, most of the commercial DFs, such as inulin and fructooligosaccharides (FOS), are soluble and have high fermentation properties similar to FODMAP (19), which can easily lead to the aggravation of symptoms in IBS patients. Therefore, it is important to develop new DFs suitable for a low FODMAP diet therapy in IBS.

In this paper, WIP was extracted by alkali-extraction and acid-precipitation method. Its structure, molecular weight were identified and its properties related to stability, digestion, viscosity, osmotic activity, adsorption and fermentation were investigated, aimed to explore the feasibility of WIP as a new DF supplement for IBS patients with low FODMAP diet therapy.

## 2. Materials and methods

### 2.1. Materials

The dried sclerotia of *W. cocos* and carboxymethyl poria cocos polysaccharide (CMP) were provided by Hunan Butian pharmaceutical Co., LTD (Hunan, China); Inulin was purchased from Beneo Orafti (Belgium); Sorbitol Assay Kit was provided by Solarbio LIFE SCIENCES (Beijing, China); Pepsin (USP, 1: 3000), Bile powder of pig (BR) and Pancreatin (BR, 1: 4000) were obtained from Yuanye Bio-Technology (Shanghai, China).

### 2.2. WIP extraction

WIP was extracted according to the reported method with some modification (10). In brief, dried sclerotia of *W. cocos* was grounded and extracted with NaOH solution (0.75 mol/L) and neutralized with HCl (1 mol/L) to obtain polysaccharide precipitation after removing fat-soluble and water-soluble molecules with petroleum ether and hot water, respectively. The crude polysaccharide was further washed by distilled water, dialyzed (molar mass truncation, 8.0 kDa) to remove inorganic salts and other impurities, then lyophilized and stored at 4°C for further analysis.

### 2.3. Structure and molecular weight identification

Total sugar content was detected by the method of phenol-sulfuric acid; Molecular weight was determined by high performance gel permeation chromatography (HPGPC) (BRT105-104-102 tandem gel column, differential detector RI-10A) as below: The WIP sample was resolved in 0.2 M NaOH solution (5 mg/mL), incubated at 120°C for 1 h, centrifuged at 12,000 rpm for 10 min, and the supernatant was filtered through a 0.22 μm microporous membrane for determination; Infrared spectrum data was determined by KBr tablet method and the absorption spectra was recorded at 4000–400 cm^−1^ wavelengths (Bruker TENSOR27 INFRARED spectrometer); UV spectral data was determined by PERSEE TU1810 UV spectrophotometer; WIP samples were dissolved in DMSO-D6 and NMR spectra was obtained with Bruker Avance-600 M spectrometer. XRD data was obtained with Bruker D8 Avance X-ray diffractometer (2θ = 5–50°, scanning speed 40°/min, step width 0.01°).

### 2.4. Thermal stability measurement

DSC data was measured by Perkin Elmer DSC8000 differential thermal analyzer (temperature range 25–500°C, heating rate 10°C/min); TGA data was measured by Perkin Elmer TGA4000 thermogravimetric analyzer (temperature range 25–600°C) (26).

### 2.5. *In vitro* digestion analysis

The method of *in vitro* digestion was simply modified on the basis of previous studies as below (27–30).

Oral digestion: Fresh saliva was donated by three healthy volunteers with no record of antibiotic use in 3 months. Saliva was collected, mixed and centrifuged then the supernatant was collected and stored at −20°C. WIP solution was made at a concentration of 1.0 mg/mL and test tubes were divided into two groups: Group A (containing 1 mL saliva and 1 mL WIP solution) and Group B (containing 1 mL saliva and 1 mL water). All groups were placed in 37°C for 15 min, then boiled for 5 min to inactivate saliva amylase and centrifuged at 4,500 rpm for 15 min to collect the supernatant.

Gastric digestion: The gastric electrolyte solution contained 3.10 g NaCl, 1.10 g KCl, 0.15 g CaCl_2_·2H_2_O, 0.6 g NaHCO_3_ per litter (pH 2.5). To prepare *in vitro* simulated gastric fluid, 1.5 mL CH_3_COONa (1 mol/L, pH 5.0) and 35.4 mg pepsin was mixed with 150 mL gastric electrolyte solution (pH 2.5). 100 mL simulated gastric fluid was mixed with WIP to make the concentration of 1.0 mg/mL, placed in a shaker at 37°C, 50 r/min. After 0, 0.5, 1, 1.5, and 2 h, respectively, 10 mL reaction solution was boiled for 5 min and the supernatant was retained after centrifugation.

Intestinal digestion: The intestinal electrolyte solution consisted of 5.4 g NaCl, 0.65 g KCl, 0.33 g CaCl_2_·2H_2_O per litter (pH 7.0). *In vitro* simulated intestinal fluid was prepared by mixing 20.0 g trypsin solution (7%, w/w), 40.0 g bile salt solution (4%, w/w) and 20 mL intestinal electrolyte solution (pH 7.0). The simulated intestinal fluid was mixed with digested gastric solution at the ratio of 10: 3 and placed in a shaker at 37°C. After 0, 0.5, 1, 1.5, and 2 h, respectively, 10 mL reaction solution was boiled for 5 min and the supernatant was retained after centrifugation.

The concentration of reducing sugar in the supernatant was determined by the DNS method.

### 2.6. Viscosity and particle size measurement

WIP, CMP and inulin were prepared into 0.1 wt% aqueous solutions, and the hydrated particle size was determined by Litesizer 500 nanometer particle size and Zeta potential analyzer. The particle size of WIP was determined by Winner 2308 laser particle size analyzer as its size was exceeded the detection range.

WIP, CMP and inulin were accurately weighed, dispersed in water to produce a suspension (1.0 mg/mL) and then tested for viscosity using TA Instruments from Waters (Discovery HR10, 40 mm parallel plat, Gap = 1,200 μm, shear rate range: 1.0 s^-1^ ~ 100.0 s^-1^) (31).

### 2.7. Water/oil-holding capacity analysis

#### 2.7.1. Water swelling capacity (WSC) analysis

WIP (0.5 g) was mixed with simulated intestinal fluid (5 mL), placed in graduated test tubes, and treated for different times. The volume changes at 0, 2, 4, 6, and 8 h were calculated respectively, and the WSC was calculated as follows:


$$WSC=\left(V1-V2\right)/W1$$

In the formula, V_1_ is the volume before expansion, V_2_ is the volume after expansion, and W_1_ is the weight of the sample (32).

#### 2.7.2. Water-holding capacity (WHC) analysis

WIP (0.1 g, W_1_) was added to 10 mL distilled water and equilibrated at 37°C for 2 h. After centrifugation at 4,800 rpm for 10 min, the residue was immediately extracted and the weight (W_2_) was determined (33). Finally, the WHC was calculated by the following equation:


$$WHC=\left(W2-W1\right)/W1.$$

#### 2.7.3. Oil-holding capacity (OHC) analysis

WIP (0.1 g, O_1_) was added to 10 mL soybean oil and equilibrated at 37°C for 2 h. After centrifugation at 4,800 rpm for 10 min, the residue was immediately extracted and the weight (O_2_) was determined (33). Finally, the OHC was calculated as follows:


$$OHC=\left(O2-O1\right)/O1.$$

### 2.8. Detection of FODMAPs adsorption capacity

0.1 g samples (WIP, CMP) were mixed with 10 mL different FODMAPs (fructose, lactose and sorbitol) solution (5 mmol/L), respectively, and incubated for 6 h at 37°C. The contents of fructose and lactose in supernatant were determined by DNS method, and the content of sorbitol in supernatant was measured by the sorbitol detection kit.


$$\mathrm{FODMAPs adsorption capacity}=\left[n2-\left(n1-n0\right)\right]/m$$

In the formula, m is the sample mass/g; n_0_ is the FODMAPs content of the blank group /mg; n_1_ is the FODMAPs content of the sample group /mg; n_2_ is the FODMAPs content of the control group/mg (34).

### 2.9. Density measurement

0.5 g samples (WIP, CMP, INU) were added into 10 mL measuring cylinder and the volume of samples were recorded. The cylinder was then tapped 100 times on the table, and the volume of samples were recorded again. Bulk density and tapped density were calculated as the ratio of weight to volume (35). Hausner’s ratio and compressibility index were calculated by the following equation:


Hausner'sratio=Tapped density/Bulk density

$$\mathrm{Compressibility index}=\frac{\mathrm{Tapped density}-\mathrm{Bulk density}}{\mathrm{Tapped density}}\times 100.$$

### 2.10. *In vitro* fermentation

#### 2.10.1. Collection and preparation of fecal inoculum

Fresh feces of three healthy donors (two women and one man, aged from 22 to 25, who had no record of antibiotic use in the past 3 months) were collected. Then, the fecal samples from three donors were mixed evenly at a mass ratio of 1:1:1, diluted 10 times with diluent (0.24 g KH_2_PO_4_, 1.44 g Na_2_HPO_4_, 8.0 g NaCl, and 0.2 g KCl per liter). The supernatant was collected after centrifugation to obtain a 10% (w/v) fecal inoculum (36).

#### 2.10.2. *In vitro* colonic fermentation

Fermentation was studied *in vitro* according to the previously described method with slight modification (25, 37). Briefly, after sterilized by UV irradiation for 12 h, 100 mg of samples (WIP, CMP, INU) were added into sterile fermentation containers, respectively. In addition, 1 mL fecal inoculum and 9 mL sterilized fermentation medium [10.0 g peptone, 4.0 g yeast extract, 1.0 g cysteine hydrochloride, 1.0 g NaCl, 0.45 g KH_2_PO_4_, 0.45 g K_2_HPO_4_, 0.05 g Hemin, 0.2 g CaCl_2_, 4.5 g MgSO_4_, 0.25 g resazurin, 10 μL Vitamin K_1_ per litter (pH 6.8)] were added, mixed, and placed in an anaerobic incubator at 37°C for 6 h. The changes of pH were measured before and after fermentation and each post-fermentation sample was collected and stored at −20°C for further analysis.

### 2.11. Post fermentation analysis

#### 2.11.1. Quantification of reducing sugar and ammonia

The content of reducing sugar in post-fermentation samples was detected by DNS method, and the phenol-sodium hypochlorite colorimetric method was used to detect the content of ammonia (38). Briefly, the broth was centrifuged after fermentation and 40 μL of supernatant was taken. Then, 40 μL of distilled water, 2.5 mL of phenol chromogenic reagent and 2.0 mL of hypochlorite reagent were added and mixed well. After heating at 37°C for 30 min, the absorbance was detected at 550 nm, and the ammonia concentration was calculated.

#### 2.11.2. Morphological analysis

Samples before and after fermentation were completely dried and fixed on aluminum posts using double-sided tape. Then, a thin layer of gold was sputtered on the surface by ion sputtering in a vacuum for 30 s. The surface and microstructure were observed with SEM (SU8020 scanning electron microscope, Hitachi, Tokyo, Japan) at 5,000× magnification.

#### 2.11.3. Gut microbiota analysis

Genomic DNAs were extracted by CTAB or SDS method. The V3-V4 region of bacterial 16S rRNA gene was amplified using 515F and 806R primers by PCR. Products were detected, purified and collected. The library was constructed using the NEBNext^®^ Ultra^™^ IIDNA Library Prep Kit, and quantified by Qubit and Q-PCR. NovaSeq6000 was used for sequencing. Paired-end sequencing was used with a length of 250 bp at each end; noise reduction was performed by DADA2; QIIME2’s classify-sklearn algorithm was used to annotate species for each ASV using a pre-trained Naive Bayes classifier; Based on the annotated results of ASVs and the character of each sample, tables of species abundance at the level of kingdom, phylum, order, family, genus and species were obtained; Unifrac distances were calculated by QIIME2 software and R software was used to plot PCA, PCoA and NMDS downscaling maps. PCA and PCoA invoked packages ade4 and ggplot2 in R software (37).

### 2.12. Statistical analysis

Statistical analysis in this study was performed using GraphPad Prism 8 (La Jolla, CA, United States). All the experiments were performed in triplicate, and the data were presented as the mean ± standard deviation (SD). Significance was assessed by one-way analysis of variance (ANOVA) and student’s t test. *P*-values <0.05 was considered to be statistically significant (*p < 0.05, **p < 0.01, ***p < 0.001).

## 3. Results and discussion

### 3.1. Structure identification and thermal stability analysis of WIP

**Figure 1:**
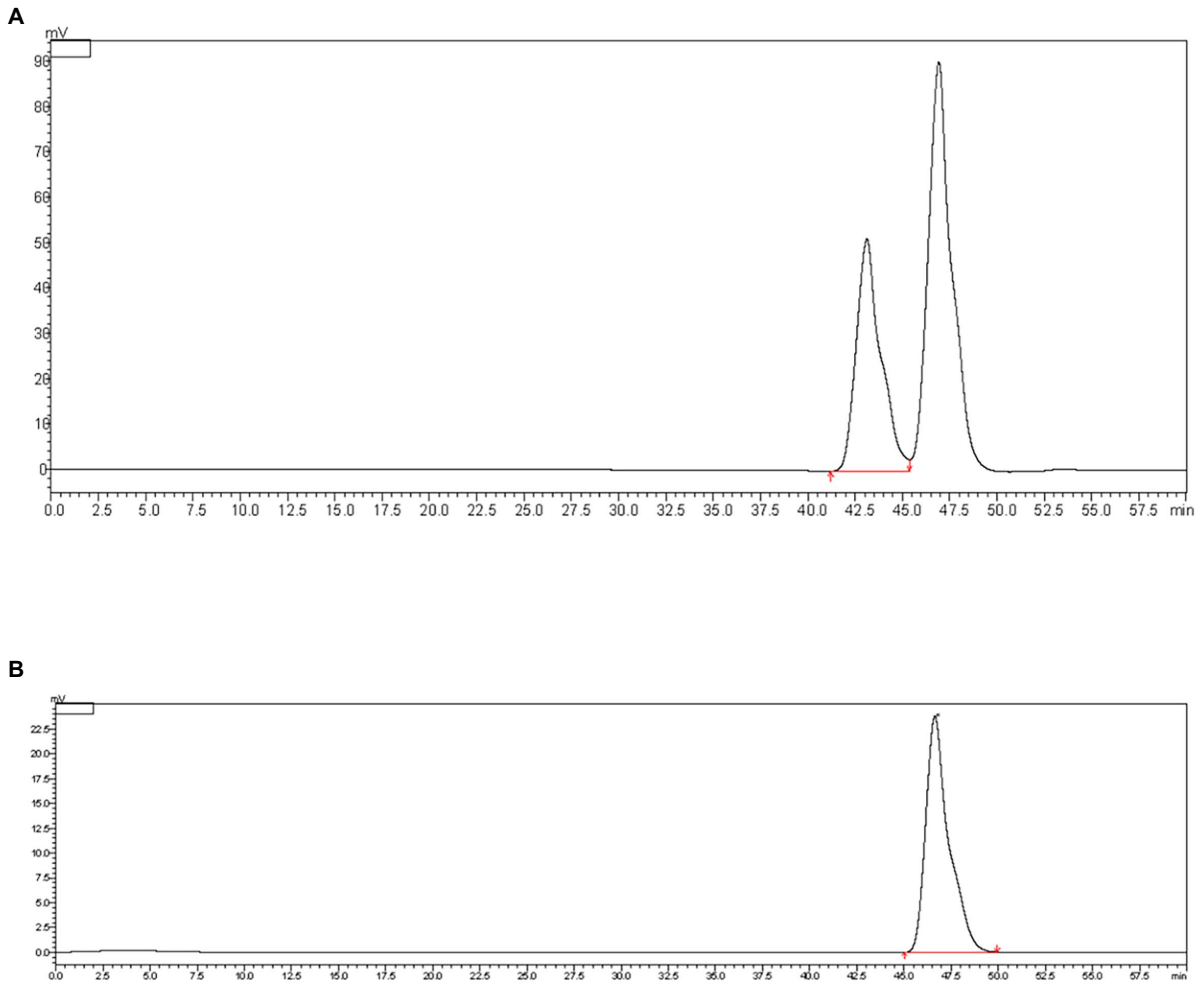
HPGPC analysis of WIP. **(A)** Molecular weight of WIP, **(B)** peak of mobile phase.

WIP is an acidic polysaccharide that is insoluble in water but soluble in alkaline conditions. Results of extraction and properties of WIP are shown in Table 1. The extraction yield of WIP was 61.6% after dialysis and freeze drying. The total sugar content detected by the method of phenol-sulfuric acid was about 90.57%. HPGPC analysis of WIP showed a single homogeneous composition and the molecular weight was 8.107 × 10^3^ Da (Figure 1). According to the dispersion coefficient of WIP, the homogeneity of its molecular weight was high and the molecular weight distribution was narrow. Compared with the results in previous study of Liu et al., the extraction yield of insoluble *Wolfiporia cocos* polysaccharide was 39.8% and the molecular weight was 4.486 × 10^6^ Da (10). In our study, the extraction yield of WIP was higher and the molecular weight was lower, which might attribute to the differences in the origin of *W. cocos* and the extraction process of polysaccharides.

**Table 1:** Properties of water-insoluble polysaccharide from *W. cocos* (WIP).

Properties	Results
Extraction yield of WIP	61.6%
Polysaccharide content	90.57%
Protein content	0
Mn	6,192
Mw	8,107
Mw/Mn	1.31

**Figure 2:**
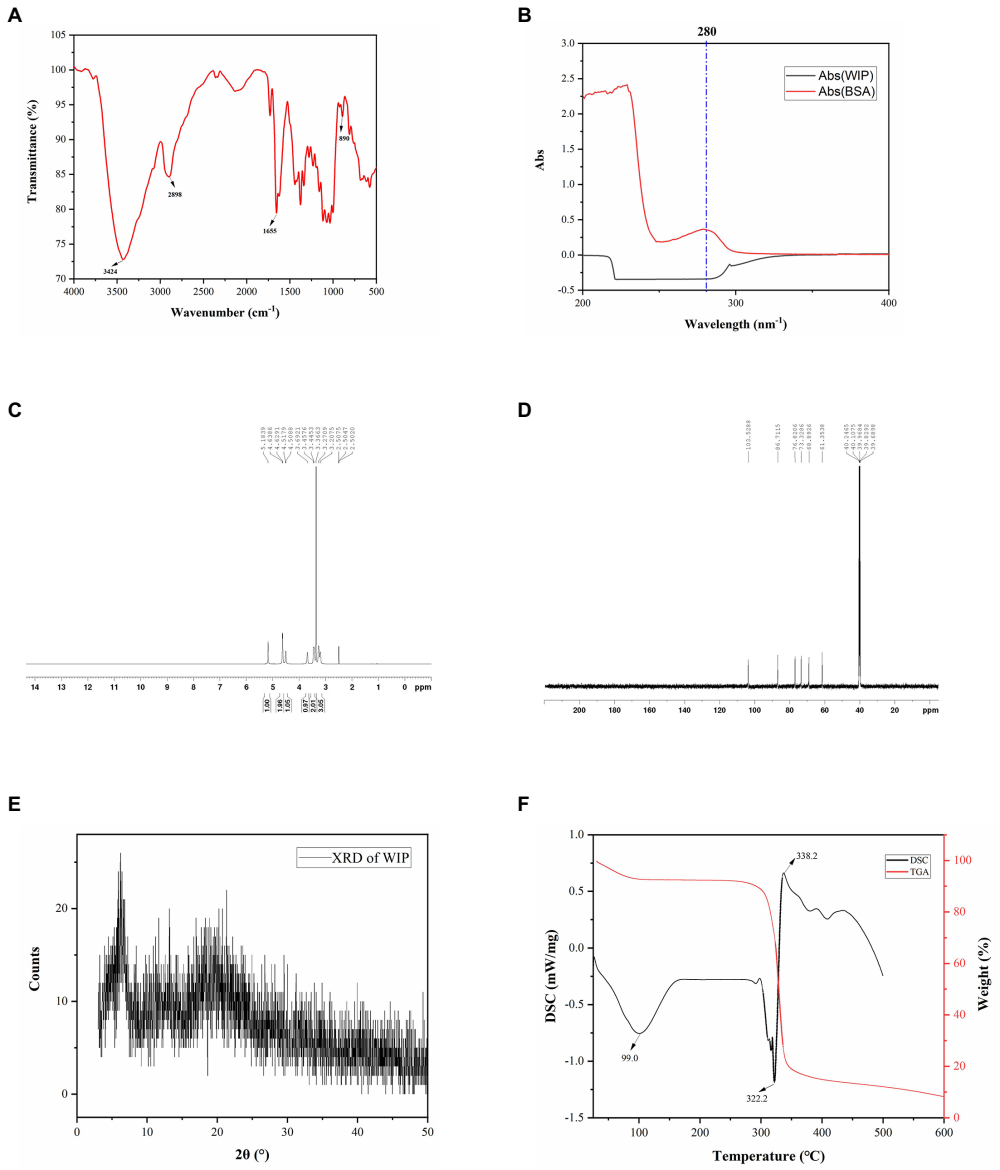
Structural characterization of WIP. **(A)** FT-IR, **(B)** ultraviolet spectrogram, **(C)** 1H spectrum in DMSO-d6, **(D)** 13C spectrum in DMSO-d6, **(E)** XRD, **(F)** DSC and TGA of WIP.

The Fourier transform infrared (FT-IR) spectrum of WIP in Figure 2A showed that the absorption peaks at 3,424 and 2,898 cm^−1^ were corresponding to the vibration of O-H and C-H; the absorption peak at 1,655 cm^−1^ was caused by an asymmetric vibration of -C=O; the absorption band centered at 1,200–1,000 cm^−1^ indicating the vibration of -C-O-C, -C-O-H; the absorption peak at 890 cm^−1^ showed the presence of β-glycosidic linkages. Based on the findings, WIP was indicated as a kind of pyranose form of sugar with β anomeric configuration.

According to the UV scanning result of WIP solution in Figure 2B, there was no characteristic absorption peak at 260–280 nm, demonstrating that WIP contained almost no protein.

In consistent with the reported study, (10) signals at 103.5/4.5, 73.3/3.27, 86.7/3.44, 68.8/3.20, 76.8/3.27, 61.3/3.69 observed in the ^1^H and ^13^C Nuclear Magnetic Resonance (NMR) spectra of WIP confirmed that the main chain of WIP was 1,3-β -glucan (Figures 2C,D).

Result of X-ray diffraction (XRD) in Figure 2E showed that the main diffraction peaks of WIP were located at 2θ = 6° and 18.3° with weak intensities, indicating that WIP was crystallized inside, and the crystallinity was very weak, which belonging to the amorphous structure (39).

Since heat treatment is unavoidable in food processing, thermal stability is an important property of polymers used in food. In the process of heating, evaporation of free and bound water occurred at first. With the increase of temperature, the disaggregation of polysaccharide chains and the fracture of C-H bonds lead to the weight loss in the thermal decomposition process. The thermogravimetric (TGA) curve in Figure 2F demonstrated that the maximum weight loss occurred in the second stage (270–350°C), which may be affected by the thermal decomposition of WIP. In the final stage (350–600°C), the weight loss rate of WIP slowed down, which may be due to the thermal decomposition of carbon (39). The Dynamic Stability Control (DSC) curve in Figure 2F showed that there was an absorption peak around 100°C, which was formed by the free water and bound water evaporated in the sample (40). The endothermic processes were at 58.8, 283.5, and 302.1°C, respectively, and the maximum peak appeared at 322.2°C. This was due to the violent thermal decomposition reaction, leading to the breakage of carbon and hydrogen chains. Yang et al. also found the similar range of weight loss in acidic polysaccharide from *Ribes nigrum L*; Arab et al. showed that polysaccharide from *Ocimum album* L. seed had familiar thermal properties (40, 41). These results proved that WIP had good processing thermal stability.

### 3.2. *In vitro* digestibility

**Figure 3:**
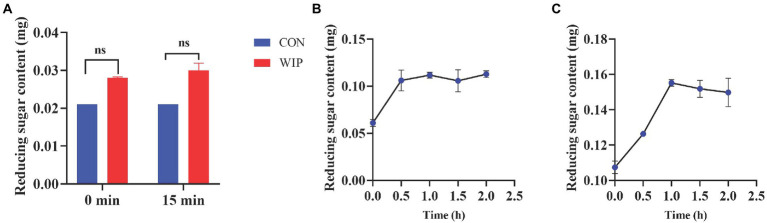
Changes in reducing sugar contents of WIP. **(A)** Oral digestion, **(B)** gastric digestion, **(C)** intestinal digestion during *In vitro* digestion.

In general, cleavage of glycosidic bonds in polysaccharides leads to an increase in the number of reducing sugars (42). In the process of WIP *in vitro* digestion, there was no significant difference in the amount of reducing sugar released during oral digestion by the Student’s t test statistical analysis (Figure 3A), indicating that WIP could hardly be hydrolysed by saliva; After gastric digestion for 2 h, the content of reducing sugar increased from 0.06 to 0.11 mg, which might be related to the partially decomposition of glycosidic bonds and formation of reducing ends in WIP under the environment of gastric acid and pepsin (Figure 3B); After 2 h of intestinal digestion, the reducing sugar content increased from 0.11 mg to 0.15 mg, probably due to the action of trypsin and bile salt (Figure 3C). In Zhu’s study, an increase in the amount of reducing sugar was observed after *in vitro* digestion, which related to the hydrolyzed effect of amylase (43). Based on the findings that only a small amount of WIP was degraded during the oral and gastrointestinal digestion process, we speculated that WIP had low bioavailability and might reach the colon mostly without being digested.

### 3.3. Viscosity and osmotic activity analysis of WIP

Studies confirmed that non-viscous insoluble fibers (such as wheat bran and cellulose) can mechanically stimulate the intestinal mucosa and promote mucus secretion (19, 44). Molecular particle size of carbohydrates determines the sensitivity to digestion, water-holding capacity and intestinal transport time (45). What’s more, Yao et al. exhibited that particle size of dietary fiber has diverse effects on *in vitro* gut fermentation rate (46).

Increased water content in the intestinal lumen is associated with smaller particle size of carbohydrates. Small molecules that are osmotically active, causing water to be reabsorbed into the colon, which stretches and softens stool. However, if infiltration occurs over time or in excess, it can cause diarrhea and may exacerbate symptoms in people with diarrhea-predominant IBS (IBS-D).

Therefore, in this part of study, we investigated the viscosity characteristics and particle size of WIP. Two kinds of water-soluble polysaccharides are selected as comparisons. One is Inulin (INU), which is rich in water-soluble dietary fiber and is often used as a high-fiber food ingredient; the other is Carboxymethyl *Wolfiporia cocos* polysaccharide (CMP), which is made by carboxymethylation modification on the basis of *WIP* to increase its solubility (47). The purpose of selecting CMP as comparison is to explore the effect of solubility and carboxymethyl structure on the functional properties of WIP.

**Figure 4:**
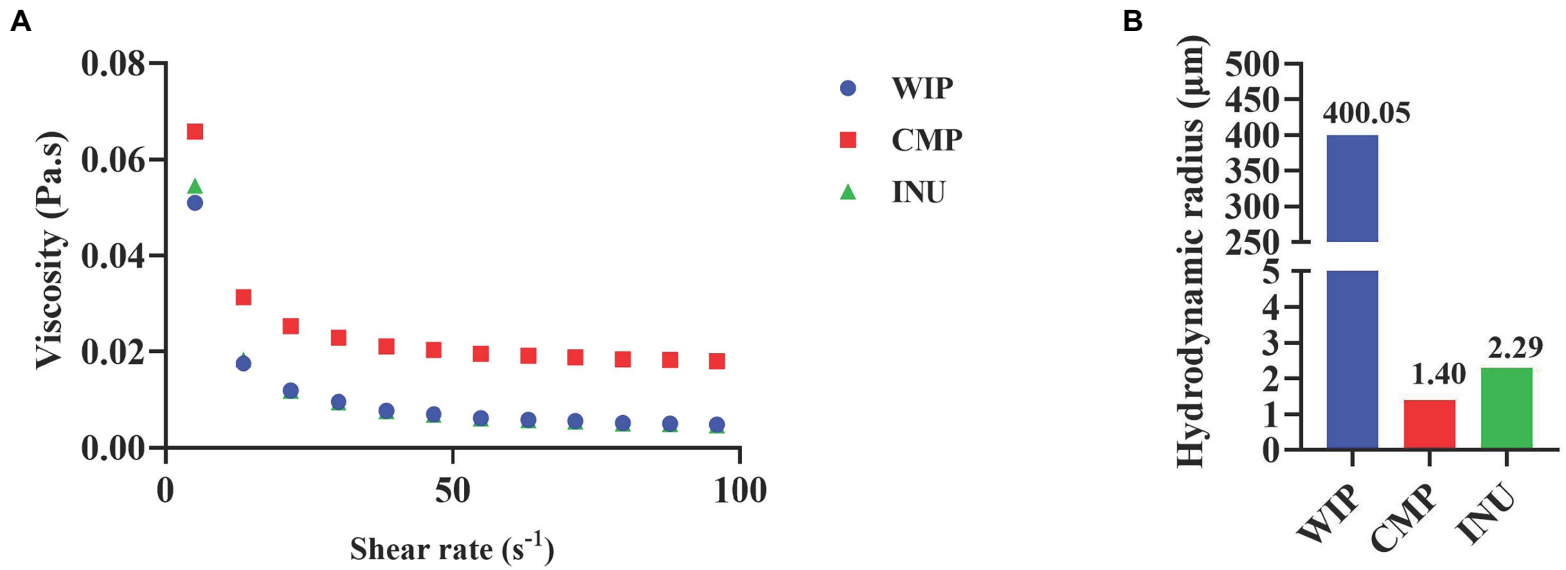
Viscosity and osmotic activity of WIP. **(A)** Viscosity, **(B)** particle size of WIP, CMP and INU.

The results of viscosity measurement in Figure 4A showed that with the increase of shear rate, the viscosity of WIP, CMP and INU decreased, indicating that the shear thinning flow occurred. Shear thinning fluid conforms to the pseudoplastic flow law, and most of the liquid foods are shear thinning fluid. WIP maintained a low viscosity with the change of shear rates, which may be related to its water-insoluble property. According to the results in Figure 4B, the particle size of WIP is larger than INU and CMP, which indicated that its osmotic activity is poor and would not cause diarrhea or other symptoms due to osmosis. Clinical study confirmed that the effect of larger insoluble fibrous particles on the colonic mucosa, such as stimulates water and mucus secretion, shortens transit time and increases stool volume for fecal excretion (48). Therefore, WIP with low viscosity and large particle size related low osmotic activity may be helpful for both IBS-D and constipated IBS (IBS-C) patients.

### 3.4. Adsorption characteristics analysis of WIP

#### 3.4.1. Water/oil-holding capacity (WHC/OHC) of WIP

Water and oil-holding capacity provides information on the pore volume of dietary fibers, which reflects their function during the intestinal transit. The hydration properties of fibers affect their metabolic activity in the intestinal tract, and the swelling effect of feces is also related to the water retention of fiber (49). Moreover, in the self-reported food intolerance survey of IBS patients, many intestinal symptoms are associated with high-fat food intake (50, 51). Therefore, supplementing the diet with ingredients that can absorb oil may relieve the symptoms of IBS patients.

**Figure 5:**
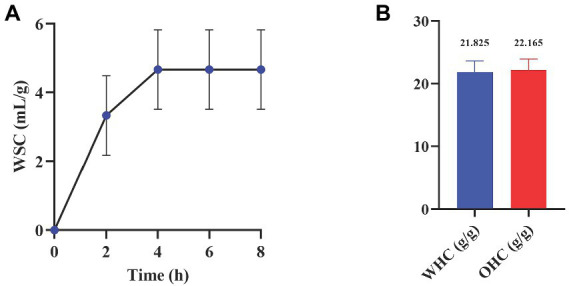
Water and oil adsorption characteristics of WIP. **(A)** WSC, **(B)** WHC and OHC of WIP.

According to the results in Figure 5A, the water swelling capacity (WSC) of WIP in simulated intestinal fluid was 5.33 mL/g. WHC and OHC of WIP were 21.825 and 22.65 g/g, respectively (Figure 5B), which were higher than reported dietary fibers extracted from sweet potato residue (52) and Foxtail millet bran (53). WHC and OHC of dietary fiber are related to particle size, surface characteristics and hydrophobicity (54). Good WHC and OHC of WIP may attribute to its fluffy texture and large particle size, indicating that WIP had potential in promoting fecal volume expansion and accelerating defecation.

#### 3.4.2. Fructose adsorption capacity of WIP

Many symptoms of IBS may be caused by the intake of poorly absorbed FODMAPs with high osmotic activity, especially fructose, lactose, and sorbitol (55). Fermentation of these molecules in the proximal colon can produce gas, leading to swelling, abdominal distension and abdominal pain symptoms (56). Some insoluble dietary fibers have been confirmed to have the property of adsorbing glucose (34, 57), thus we carried out the adsorption experiment of WIP on fructose, lactose and sorbitol.

Comparing with the control and CMP group, the fructose content in the solution was significantly reduced after the WIP adsorption treatment. Therefore, we concluded that WIP has some ability to adsorb fructose and the adsorption capacity was 0.17 mg/g based on the data in Table 2. However, it had no obvious adsorption effect on lactose and sorbitol (Table 2).This specific adsorption effect may be due to the lower molecular weight (fructose Mw = 180.16, lactose Mw = 342.3, sorbitol Mw = 182.17) and higher solubility of fructose (fructose 3.75 g/mL, lactose 0.216 g/mL, sorbitol 2.2 g/mL), which had a higher diffusion rate in the solution system and easier to be adsorbed by WIP. The results confirmed that WIP had the ability to adsorb FODMAP components and might reduce the occurrence of IBS symptoms. While CMP had poor adsorption capacity compared with WIP, we supposed that it related to its tighter texture, smaller particle size and higher bulk density. Therefore, we studied their density characteristics in the next chapters.

**Table 2:** The contents of FODMAPs after the WIP adsorption treatment.

	Fructose content (mg)	Lactose content (mg)	Sorbitol content (mg)
CON	0.512 ± 0.007	0.694 ± 0.011	9.81 ± 0.117
WIP	0.495 ± 0.001*	0.697 ± 0.009	8.40 ± 0.038
CMP	0.503 ± 0.008	0.680 ± 0.012	8.60 ± 0.020

(Means ± standard deviation. *After the data indicates a significant change *p* < 0.05).

### 3.5. Density characteristics of WIP

It has been reported that the mechanism of adsorption capacity of dietary fiber is due to the absorption or inclusion of small molecules in its internal structure (34, 58). The bulk density and tapped density provide insight into the particles accumulation, arrangement and the compaction distribution of the material. Bulk and tapped densities, Hausner’s ratio and compressibility index depend on particle shape, distribution of size and tendency to stick together (59). To explore the mechanism related to the adsorption property of WIP, we measured its density-related indicators.

**Figure 6:**
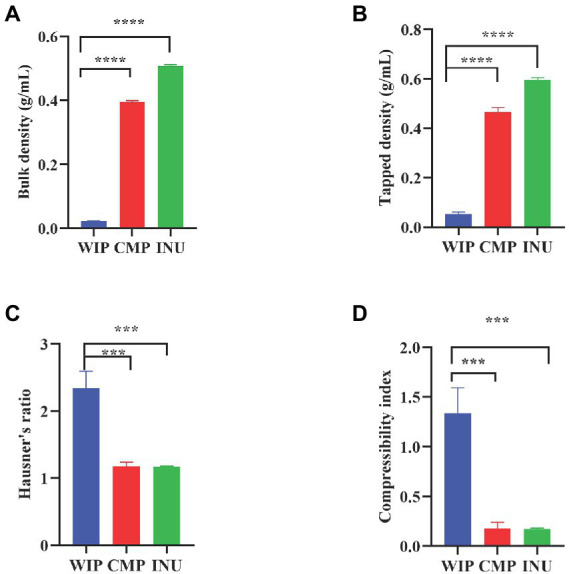
Density characteristics of WIP. **(A)** Bulk density, **(B)** Tapped density, **(C)** Hausner’s ratio and **(D)** compressibility index of WIP, CMP, and INU. ***p < 0.001 and ****p < 0.0001.

WIP had the lowest bulk and tapped densities compared with CMP and INU (Figures 6A,B), suggesting a looser porous texture which could explain its ability to absorb fructose. Normally, Hausner’s ratio greater than 1.60 indicates that the material has very poor fluidity; simultaneous compressibility index value >38% also leads to very poor fluidity (60). According to the results of Hausner’s ratio in Figure 6C, it can be concluded that comparing with CMP and INU, the fluidity of WIP is poor, which may be related to its larger particle size. Interstitial cavities (external porosity) result in high compressibility and the more holes in a matrix, the higher of its compressibility (61). Figure 6D showed that WIP had a higher compressibility index than CMP and INU, which might explain its ability to adsorb water, oil and fructose. In addition, Hausner’s ratio is used to reflect the flow capacity of powder. The fluidity of the powder affects the choice of formulation type and surface morphology in its applications. For example, powders with poor fluidity often have rough surfaces that are not easily dispersed and should not be made into granules. Therefore, granulation should be avoided in applications of WIP.

### 3.6. *In vitro* fermentation characteristics of WIP

#### 3.6.1. Effect of WIP on pH, reducing sugar and ammino content

*In vitro* fermentation are widely involved in evaluating the ability of gut microbiota to metabolize various foods under anaerobic conditions, providing theoretical bases for the development of functional foods (42). Therefore, it is necessary to study the metabolic properties of fibers by *in vitro* fermentation, and to select the dietary supplement ingredients that are expected to be used in the low FODMAP diet therapy. Due to the size limitation of the anaerobic incubator in this rapid fermentation model, it was difficult to place a stirring device in it, so we simply vortex-mixed the samples prior to performing the *in vitro* fermentation. In our future study, a stirring device will be set up in a new anaerobic incubator.

In our early exploration of the prebiotic activity of WIP, we found that it was not easy to be utilized by *Lactobacillus rhamnosus GG* (Supplementary Figure S1). Therefore, we speculated that WIP had low fermentation activity and might be a kind of dietary fiber suitable for IBS patients.

**Figure 7:**
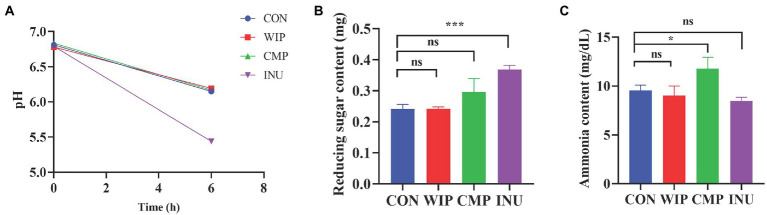
Effect of WIP, CMP and INU on **(A)** pH value, **(B)** reducing sugar content, and **(C)** ammonia content.*p < 0.05 and ***p < 0.001.

Fermentation of polysaccharide by the gut microbiota produces acidic end products such as lactic acid and short-chain fatty acids, which lead to a decrease in the intestinal pH. By monitoring the pH changes during the fermentation process, we found significant drop in the pH values of the WIP, CMP and INU groups after fermentation (Figure 7A). There was no significant difference among the WIP, CMP and CON groups, while pH in the INU group showed the greatest decline, which indicated that the fermentation performance of WIP and CMP was lower than INU. So et al. proved that the total gas production has negative correlation with post-fermentation pH (25). Based on the change trend of pH, it was speculated that the gas production of WIP should be much lower than INU. Therefore, WIP was less likely to cause abdominal distension and more suitable for IBS patients.

Traditional *in vitro* fermentation models usually run for 24 or 48 h, which lacks clinical relevance to irritable bowel syndrome, where symptoms are more attributable to the rapid production of gas over a short period of time, so we opted for a short, rapid fermentation model (25, 37). In the initial fermentation, the gut microbiota hydrolyzed the glyosidic bonds of the polysaccharides to release reducing sugars as a carbon source and an increase in reducing sugar content usually occurs at the beginning of fermentation. Guo et al. (42) mentioned that during the fermentation process, the intestinal flora utilized polysaccharides from *Clitocybe squamulosa* continuously, resulting in the breakage of glyosidic bond and the exposure of reducing ends, producing short-chain reducing sugars. When the fermentation time increased from 0 to 6 h, the reducing sugar content increased from 0.50 ± 0.03 to 0.80 ± 0.02 mg/mL. A significant increase in the amount of reducing sugars during fermentation was also observed in the study of Liu et al. (62). In our experiment, there was no difference among the WIP, CMP and CON group after fermentation, while the amount of reducing sugar in INU group increased significantly (Figure 7B), confirming that WIP was lower fermented by the gut microbiota. In addition, the solubility and carboxymethyl structure would not affect its low fermentation characteristics. It is worth noting that the determination of ammonia content showed no difference among the WIP, INU and CON group, while the ammonia content in CMP group increased lightly (Figure 7C), indicating that the CMP group had a higher level of protein fermentation. This may be due to the differences in solubility and viscosity between WIP and CMP (38).

These results showed that compared with INU, WIP was more suitable for IBS patients, and the presence of carboxymethyl structure also had impact on its fermentation performance.

#### 3.6.2. Morphological changes of WIP after fermentation

**Figure 8:**
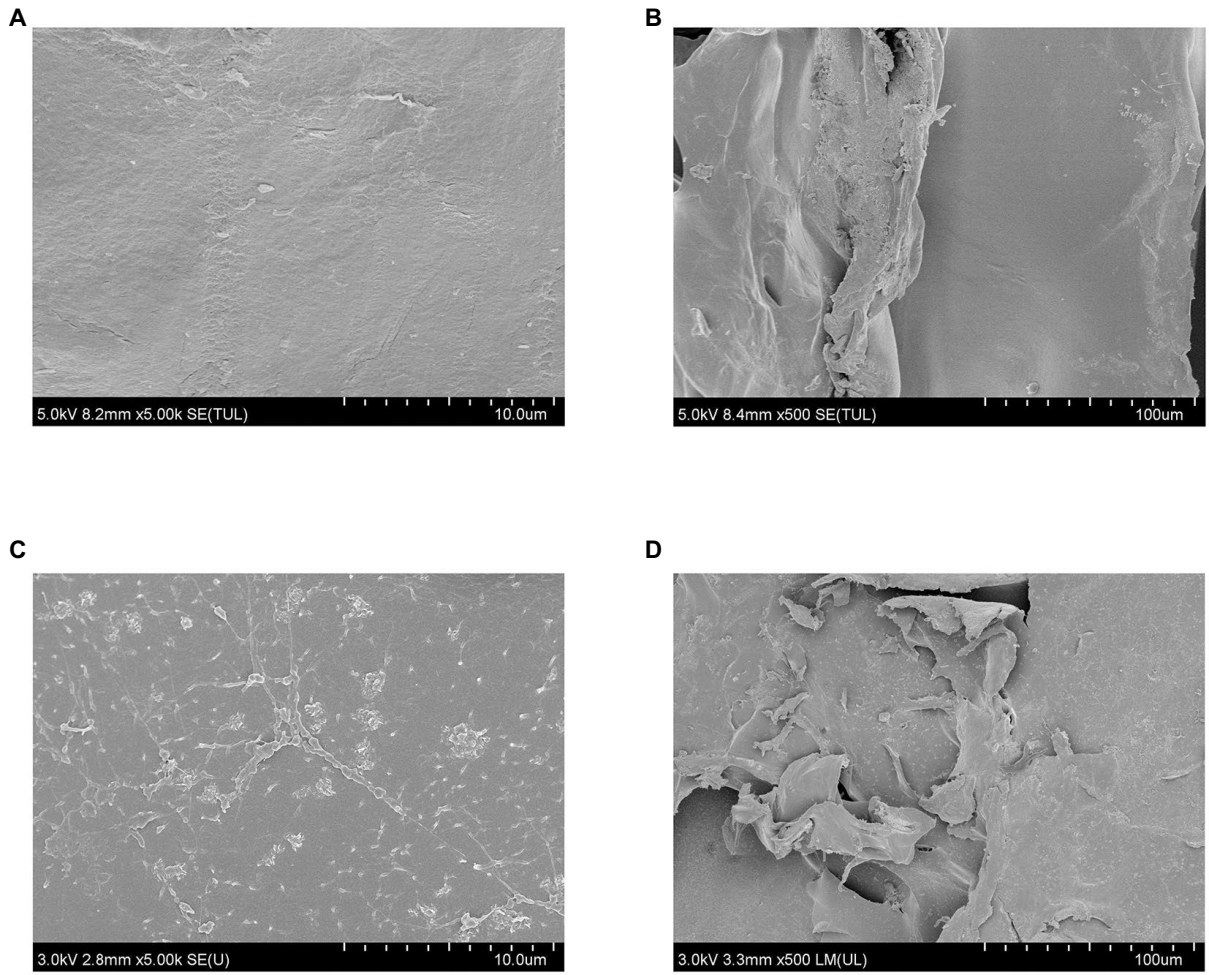
Morphological changes of WIP during *in vitro* fermentation. **(A,B)** SEM of WIP before fermentation, **(C,D)** SEM of WIP after fermentation.

Porosity determines the extent that enzymes or bacteria can diffuse into the fiber, which will greatly affect its fermentability (48). Low porosity of the food matrix in the large intestine of humans usually hinders its fermentative degradation. According to the scanning electron microscope (SEM) images, the WIP was a kind of flat and dense sheet with few pores (Figures 8A,B). After fermentation, its structure remained intact, with increasing roughness of the surface (Figures 8C,D). Guillon et al. pointed out that the pore volume accessible to gut bacteria in beet fiber affected its fermentability, which consistent with our findings (63). Hence, we speculated that due to the low porosity, flat and dense surface of WIP, it had low fermentation characteristics and might be suitable for IBS patients.

#### 3.6.3. Effect of WIP on gut microbiota composition

Gut microbiota plays an important role in maintaining host health, and dysbiosis of intestinal flora associated with increased susceptibility to disease (64). In this study, we collected stool samples from healthy donors, sequenced the fermented flora, combined with relevant literature to explore whether WIP has a modulating effect on the flora associated with the development of IBS. It is carried out based on the published papers which used *in vitro* fermentation of healthy human fecal samples to demonstrate the ability of the substrates to improve the disease. For example, in the study of Ge et al. (37), stool from a healthy donor was used to explore the potential of bamboo dietary fiber to improve obesity based on its modulation on the obesity-related flora. Although healthy human fecal samples were used in current studies to illustrate the modulatory effect of the samples on the disease-associated flora, this is limited by the fact that the samples cannot be guaranteed to have the same effect after changes in the intestinal microecology of patients. Therefore, we are working with Nanjing Hospital of Chinese Medicine Affiliated to Nanjing University of Chinese Medicine to collect stool samples from IBS patients to verify the effect of WIP on the regulation of IBS related intestinal flora in the future.

**Figure 9:**
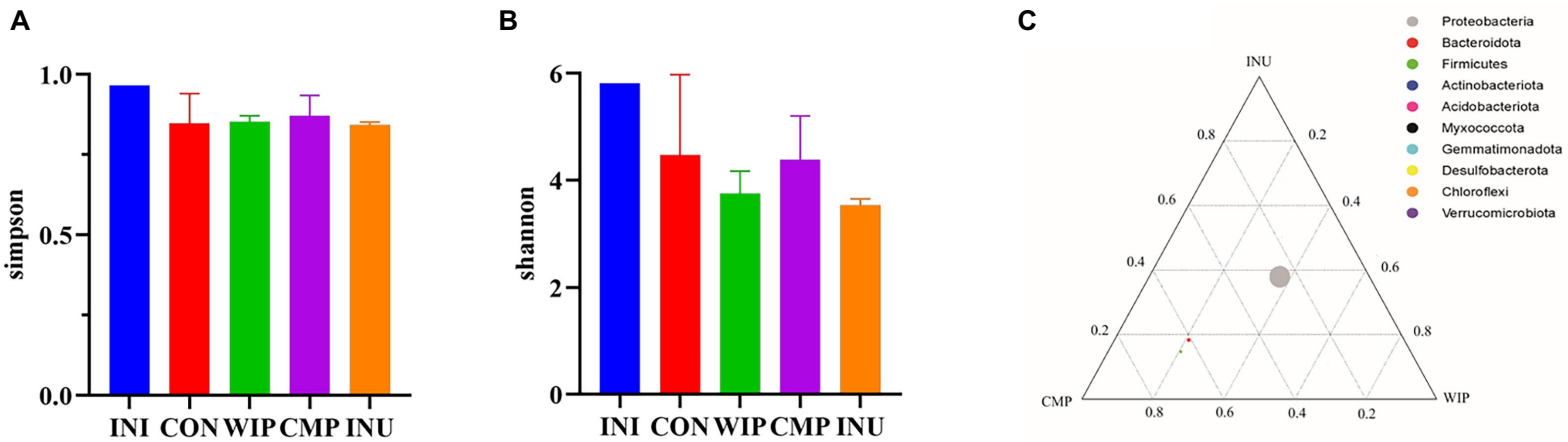
Diversity and evenness analysis of gut microbial communities. **(A)** Simpson index, **(B)** Shannon index, and **(C)** ternary plot in WIP, CMP, and INU.

Changes in gut bacterial composition can be determined by high-throughput analysis of 16S rRNA of fermentation broth. Simpson index can be involved to evaluate the diversity and evenness of gut microbial communities. The better evenness of species, the greater of the Simpson index. The Shannon index is widely used to describe the uncertainty and disorder of the individual species. The higher the uncertainty, the higher the diversity. Bacterial diversity may be affected by different algorithms, leading to different results (65). Our results in Figure 9A showed that the Simpson index of each group was large and there was no obvious difference, indicating that the species diversity and uniformity in each group was good. The Shannon index was slightly down-regulated in all groups after fermentation, while the changes were not significant (Figure 9B). It was reported that *Bacteroidetes, Firmicutes*, and *Proteobacteria* can degrade complex and indigestible polysaccharides (66). Ternary analysis in Figure 9C showed that *Bacteroidetes*, *Firmicutes*, and *Proteobacteria* were dominant phyla among all the groups, indicating they were the main bacterial groups that degraded WIP, CMP, and INU.

**Figure 10:**
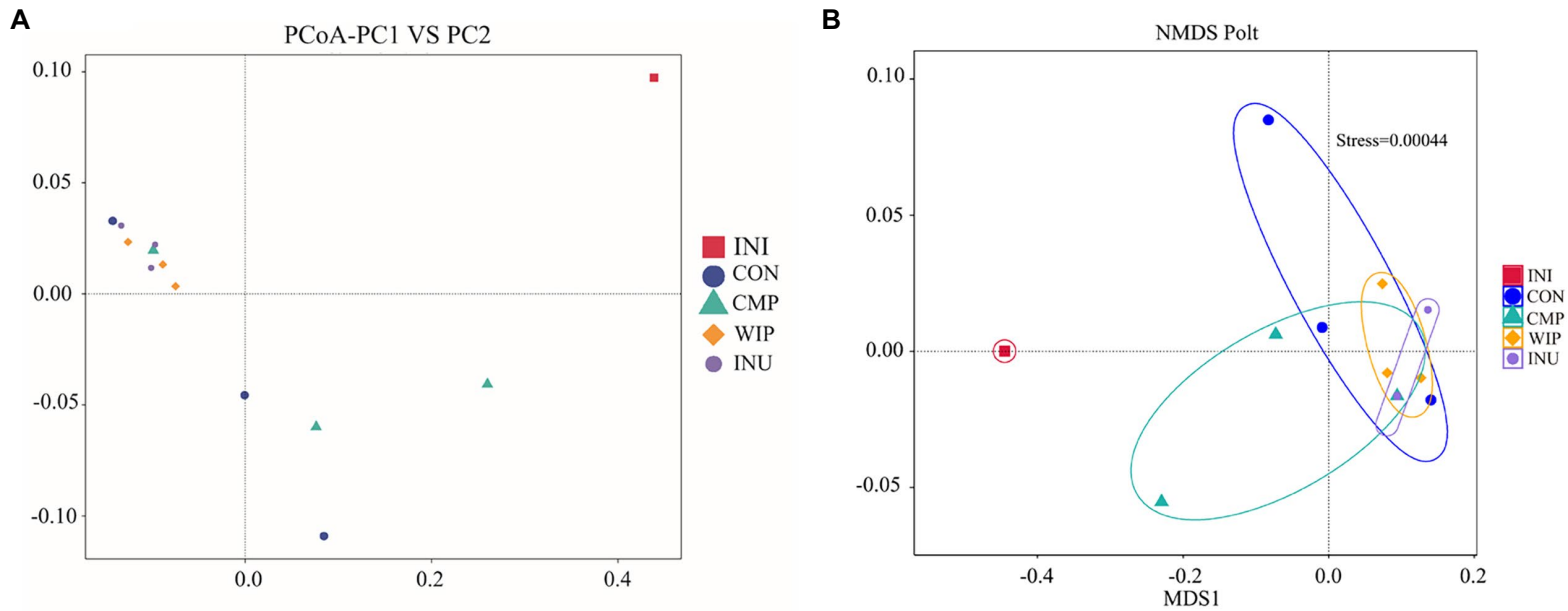
β-diversity analysis of gut microbial communities. **(A)** PCoA, **(B)** NMDS of WIP, CMP, and INU.

Differences among the gut microbial communities in each group were analyzed according to the β-diversity. There were statistical differences among the groups after 6 h of fermentation, and the principal coordinate component (PCoA) PC-1 exhibited the maximum data change of 88.34%. All groups were far away from the initial flora before fermentation (INI group), indicating that the diversity of bacterial community changed after *in vitro* fermentation (Figure 10A). Non-metric multidimensional scale (NMDS) was also used to assess the similarity of microbial communities among different groups. In Figure 10B, the distances between INI and other groups were long, confirming that the bacterial communities in WIP, CMP and INU group changed significantly after fermentation.

**Figure 11:**
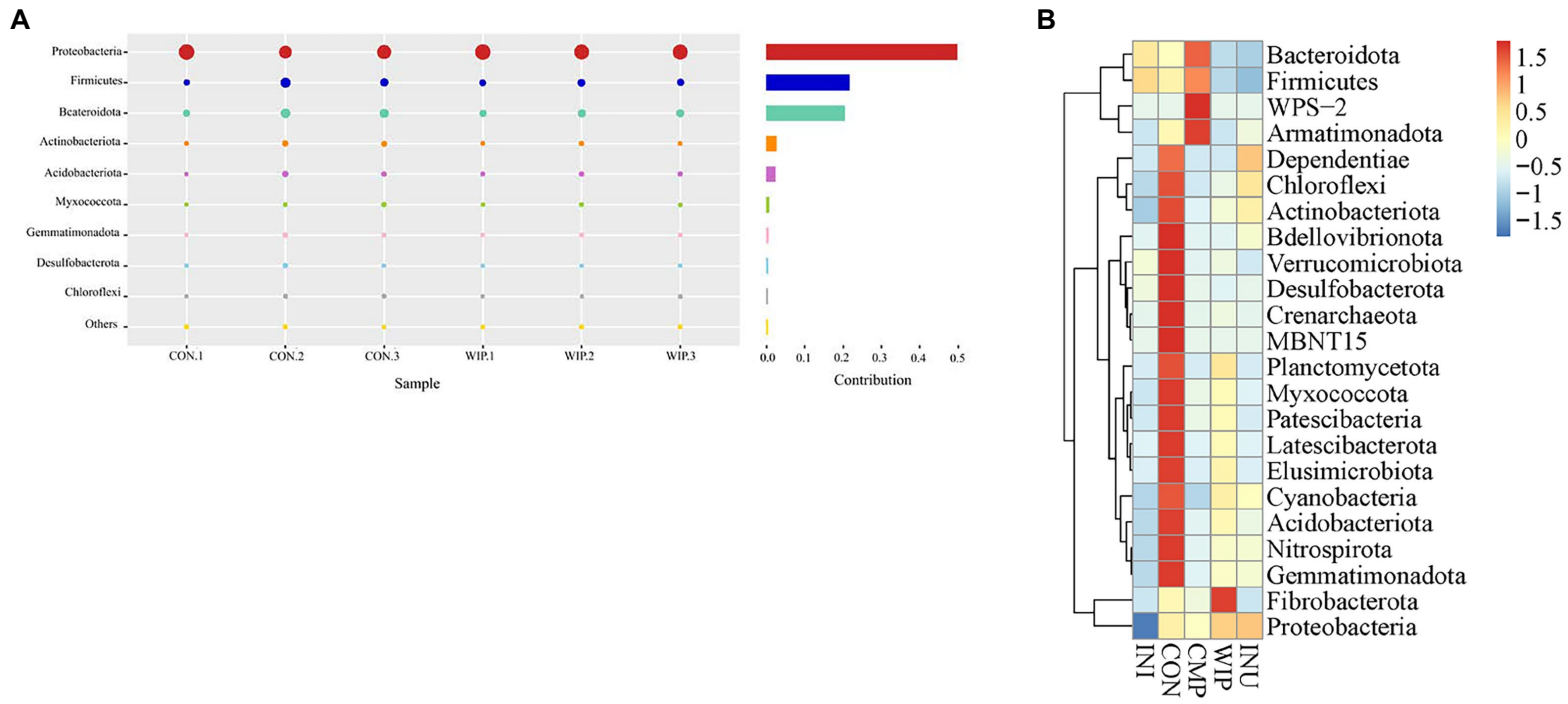
Contribution of species on gut microbial communities. **(A)** Simper analysis of WIP, **(B)** Heatmap analysis of WIP, CMP, and INU at the phylum level.

In gut microbiota composition analysis, Simper can be used to analyze the contribution of specific species in the change of community composition, and select species with significant differences among different communities (67, 68). According to the result of Simper plot in Figure 11A, the top three differentially contributing phyla between WIP and CON were Proteobacteria, Firmicutes and Bacteroidetes, respectively. Species belonging to the clade of *Fibrobacterota* possess high proportions of genes encoding carbohydrate-active enzymes (CAZy). Hence, *Fibrobacteriaceae* is particularly suitable for degrading dextran, cellulose, mannan, acacia, xylan, xyloglucan, chitin, starch and pectin (69). The microbiota heatmap in Figure 11B exhibited the highest abundance of *Fibrobacteriaceae* in the WIP group, suggesting that it might be the key microorganisms in WIP degradation.

**Figure 12:**
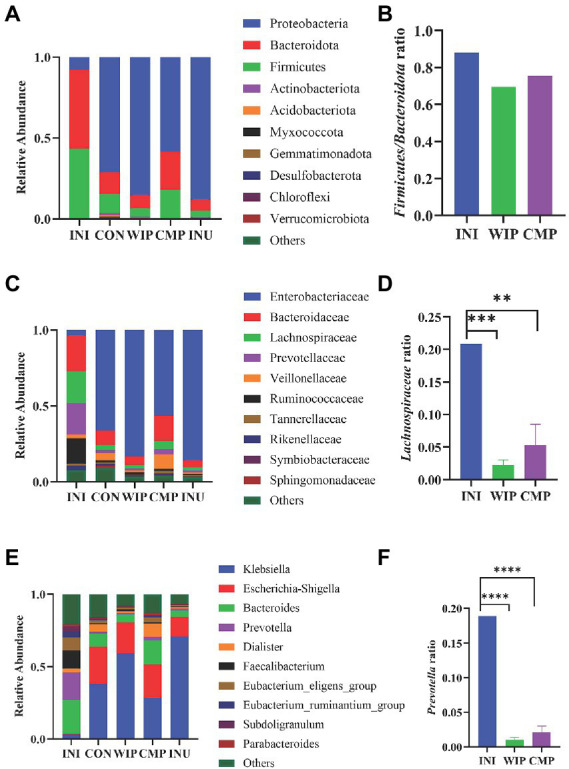
Effect of WIP on gut microbiota composition. **(A)** Phylum level, **(B)** Firmicutes/Bacteroidetes ratio, **(C)** family level, **(D)** Lachnospiraceae ratio, **(E)** genus level, and **(F)** Prevotella ratio. **p < 0.01, ***p < 0.001, and ****p < 0.0001.

Intestinal flora imbalance is closely related to the pathogenesis of IBS. We further analyzed the relative abundance of species at the phylum, family and genus levels, respectively, and identified the microorganisms with obvious changes. Yao et al. demonstrated that the proportion of *Firmicutes*/*Bacteroidetes* (F/B) in stool samples from IBS patients was significantly increased (70). As shown in Figures 12A,B, the F/B ratio in WIP group was down-regulated compared with the INI group, suggesting that WIP had the potential to alleviate F/B imbalance in IBS patients.

As a potential marker of intestinal flora imbalance, *Lachnospiraceae* is associated with the occurrence of many diseases. Significant increase in *Lachnospiraceae* abundance was reported in IBS-D patients (71). We found that the proportions of *Lachnospiraceae* decreased in both WIP and CMP groups, which was more obvious in WIP group (Figures 12C,D). Thus, WIP may regulate the excessive proliferation of *Lachnospiraceae* in IBS patients.

The change of gut microbiota is related to the increase of visceral hypersensitivity. Previous studies have shown that the high abundance of *Prevotella* may be positively correlated with the high risk of IBS-D (72–74). The genus level abundances shown in Figures 12E,F demonstrated that the abundance of *Prevotella* was significantly down-regulated in the WIP and CMP groups after fermentation. Based on the results, we speculated that WIP had the high potential to regulate IBS related gut microflora.

## 4. Conclusion

Taken together, the most striking finding in our study was that the characteristics and properties of WIP extracted from *W. cocos*, including stability, digestion, viscosity, osmotic activity, adsorption and fermentation, meet the requirements of dietary fiber suitable for a low FODMAP diet therapy in IBS and related mechanisms were also explored. Moreover, it is worth noting that WIP regulates IBS associated gut microbiota effectively, such as the abundance of Lachnospiraceae and Prevotella. Further studies are ongoing to verify the efficacy of WIP *in vivo*, which is necessary to evaluate its potential for use in intestinal disease prevention and gut health improvement with specific mechanisms. In the future, randomized clinical controlled trials will be carried out to develop WIP as a dietary supplement for IBS patients with low FODMAP diet therapy.

## Data availability statement

The original contributions presented in the study are publicly available. This data can be found at: https://www.ncbi.nlm.nih.gov/bioproject/PRJNA911433.

## Author contributions

XY: conceptualization, formal analysis, data interpreted and curation, methodology, investigation, writing – original draft, and visualization. SL and YF: investigation, validation, and data interpreted. CC: methodology and data curation. YZ: writing – review and editing. SC: project administration, funding acquisition, supervision, and writing – review and editing. All authors have read and agreed to the published version of the manuscript.

## Funding

This study was funded by the National Key R&D Program of China (2022YFF1100302), the Science and Technology Program of Jiangsu Province (Key Technologies in Agriculture and Rural Areas) (BE2022310), the Key R&D Program of Shandong Province (2021SFGC1202 and 2021SFGC1205), the Nanjing Research Center for Infectious Diseases of Integrated Traditional Chinese and Western Medicine (YBZX2022), and the National Innovation and Entrepreneurship Training Program for Undergraduate.

## Conflict of interest

The authors declare that the research was conducted in the absence of any commercial or financial relationships that could be construed as a potential conflict of interest.

## Publisher’s note

All claims expressed in this article are solely those of the authors and do not necessarily represent those of their affiliated organizations, or those of the publisher, the editors and the reviewers. Any product that may be evaluated in this article, or claim that may be made by its manufacturer, is not guaranteed or endorsed by the publisher.
