# Supplementary material for: Characteristics and properties of a polysaccharide isolated from Wolfiporia cocos as potential dietary supplement for IBS
Source: Front Nutr. 2023 Mar 27;10:1119583. doi: 10.3389/fnut.2023.1119583 (PMC10083290; doi:10.3389/fnut.2023.1119583)
Supplement: Supplementary file 1 [file Data_Sheet_1.DOCX]

Supplementary Material

Characteristics and properties of a polysaccharide isolated from *Wolfiporia cocos* as potential dietary supplement for IBS

Xuan Yang^1^, Shun Lu^1^, Yuhan Feng^1^, Chongjiang Cao^1^, Yanliang Zhang^2,3^, Shujie Cheng^1,3*^

^1^ Department of Food Nutrition and Safety/National R&D Center for Chinese Herbal Medicine Processing Technology, School of Engineering, China Pharmaceutical University, Nanjing, China

^2^ Nanjing Hospital of Chinese Medicine Affiliated to Nanjing University of Chinese Medicine, Nanjing, P. R. China

^3^Nanjing Research Center for Infectious Diseases of Integrated Traditional Chinese and Western Medicine, Nanjing, P. R. China

*** Correspondence:**Shujie Cheng

E-mail: scheng@cpu.edu.cn

**Figure S1. Effect of WIP on reducing sugar content after fermentation.**

*Lactobacillus rhamnosus GG* (*LGG*) was incubated in the culture medium (10.0 g Peptone, 5.0 g Beef extract, 4.0 g Yeast extract, 20.0 g Glucose, 2.0 g K_2_HPO_4_, 2.0 g Ammonium hydrogen citrate, 5.0 g CH_3_COONa, 0.2 g MgSO_4_, 0.05 g MnSO_4_, 1.0 g Tween 80 per litter) at 37°C for 12 h and then centrifuged at 8000 rpm for 10 min. The precipitate was washed and dispersed in sterile saline to make the value of OD 600 around 0.35 (10^8^-10^9^ cfu/mL). WIP was mixed with bacterial suspension at a ratio of 1:10 and incubated in a shaker at 37°C. The fermentation solutions were taken for reducing sugar content determination after 24 h and 48 h, respectively.


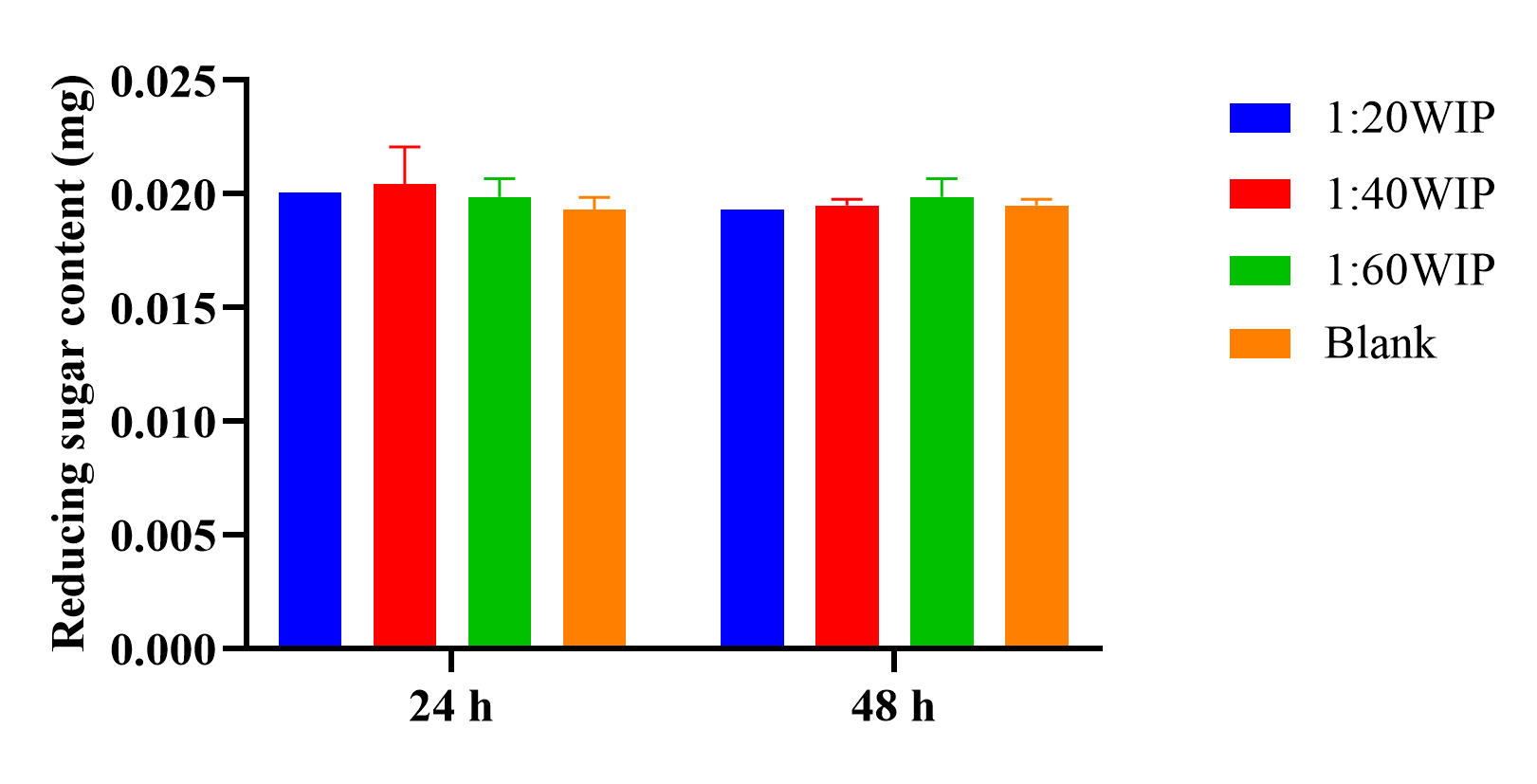


**Figure S1. Effect of WIP on reducing sugar content after fermentation.**
